# Natural Bioactive Compounds: The Way Shown by Professor Maurizio Battino and His Group in an Italian Cutting-Edge Laboratory

**DOI:** 10.3390/ijms17071038

**Published:** 2016-07-05

**Authors:** International Journal of Molecular Sciences Editorial Office

**Affiliations:** MDPI AG, Klybeckstrasse 64, CH-4057 Basel, Switzerland; ijms@mdpi.com

Maurizio Battino, Ph.D., Associate Professor of Biochemistry in the Department of Clinical Sciences, Faculty of Medicine, Università Politecnica delle Marche (Italy), is the Director of the Centre for Health and Nutrition, Universidad Europea del Atlantico (Santander, Spain) and Director of Nutrition and Health projects and Master courses at FUNIBER on-line platform (Barcelona, Spain). His research group, the Bioenergetics Group, investigates a way of mitigating disease processes through the correct use of specific foods (mainly berries and dietary fats) and of their bioactive compounds. He undertook a B.Sc. in Bologna, Ph.D. in Catania (Italy) and post-doc in Granada (Spain); he obtained a M.Sc. in the International Communication Technology in Medicine (Ancona, Italy) and was awarded with a Doctor Honoris Causa degree by the University of Medicine and Pharmacy “Carol Davila” Bucharest (Romania). He currently reviews scientific articles for over three dozen peer-reviewed journals, serves as the Editor-in-Chief for the *Journal of Berry Research*, *Mediterranean Journal of Nutrition & Metabolism* (IOS Press) and *Diseases* (MDPI) as the Associate Editor for *Molecules* (MDPI) and in the editorial board of *Food Chemistry* (Elsevier), *Plant Food for Human Nutrition* (Springer), *Nutrition and Aging* (IOS Press), and the *International Journal of Molecular Sciences* (MDPI).

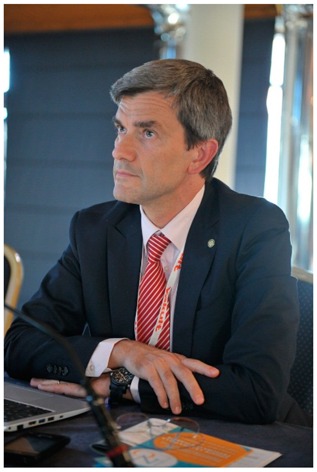

Dr. Maurizio Battino

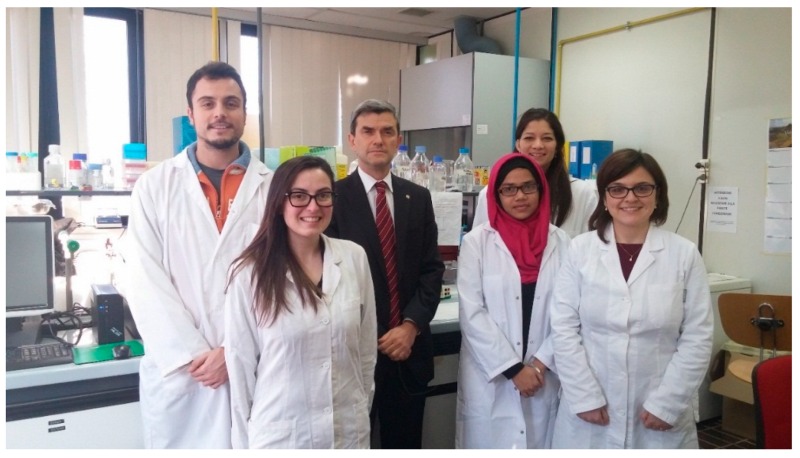

Dr. Maurizio Battino’s Research Team

Several research projects are currently being undertaken related to the definition of the role of antioxidants and bioactive compounds present in different food matrices (strawberries, honey and oil) on oxidative stress and on the modulation of several genes involved in antioxidant defenses, metabolism, cell survival and proliferation, inflammation and related disorders (fibromyalgia, periodontal disease, metabolic syndrome, etc.). The experimental models on which the group has worked and currently works include both in vitro models (fibroblasts, hepatoma cells, breast cancer cells, hypercholesterolemic cells, macrophages and adipocytes) and in vivo models (mice, rats and humans). Targeted diseases are those related directly with mitochondrial impairment (e.g., fibromyalgia) and/or inflammation processes and oxidative stress including metabolic syndrome, cancer, atherosclerosis and periodontal diseases.

Dr. Battino has more than 25 years of experience in bioenergetics and in food research with special emphasis on the role of natural antioxidants and his studies are documented in more than 250 peer-reviewed research articles with h-index = 51 according to Google Scholar MyCitations or h-index = 41 and 39 according respectively to Scopus and ISI Web of Science; he has also co-edited several books and special issues.

We are honored to carry out this interview with Dr. Battino, in which Professor Maurizio Battino shared his experience in the field of natural bioactive compounds, as well as his opinions on open access publishing.

**1.**  **Professor Battino, as is well-known to us, you are a Highly Cited Researcher (Thomson Reuters) in the category of Agricultural Sciences. Do you remember how and when you first became interested in this field?**

I think that a premise is needed: “Agricultural Sciences” category for Thomson Reuters comprises also “Nutrition”; this is why I received this award in the above category. As far as my “Nutrition” research interest are concerned, I began about 25 years ago when for the first time I studied the effects of virgin olive oil intake on mitochondrial functions and properties (see for example [1,2,3,4]).

**2.**  **Which key people influenced you?**

I am very indebted firstly to my “*maestro*” in Italy, Prof. Giorgio Lenaz who at the University of Bologna at early 1980s introduced me to the exciting world of bioenergetics and biochemistry and also to my Spanish mentor, Prof. Josè Mataix, who at the Institute for Food Technology at Granada University opened my mind to several nutritional aspects.

**3.**  **What was your feeling when you were announced as being a Highly Cited Researcher?**

I could not believe it: I thought it was a SPAM when I received the first email from Thomson Reuters and a joke from some friends when I received the second.

**4.**  **Interesting! As a prestigious expert, do you have any suggestions to young scientists?**

Have (scientific) dreams, defend them and ask your mentors to give you the possibility to demonstrate if your idea is valuable. I have always given a chance to my Ph.D. students and post-docs to support their theories even if they contrasted with my ideas: I was right! This achievement demonstrates I was right and now it is because of their challenges that we are a Highly Cited group.

**5.**  **You have studied natural bioactive compounds for more than 25 years. Which discoveries in this field would you say were the most important?**

Firstly, the recognition of the involvement and the role of antioxidants as possible tools for improving the most known and diffused chronic diseases mainly those related with low grade inflammation; on this topic also my group and co-workers have substantially contributed [5,6,7,8,9,10,11,12]. But, even more important I consider could be the evidence and the most recent message that natural antioxidants may reveal a plethora of other activities not directly or not always linked to their actual antioxidant properties and nonetheless their role is limited: just the opposite! In the last 10 years, it has been widely discussed and demonstrated [13,14,15,16]. They act at different level via not-antioxidant driven processes and their role is often essential, for cell welfare and our healthy status. Here, again, our investigations have substantially contributed to this view and they are currently considered as masterpieces in the world-wide discussion on this topic [17,18,19,20,21,22,23].

**6.**  **Can you outline what the most important ongoing projects are in your group?**

We are currently focusing upon AMPK pathways’ modifications in cancer cells [23,24,25] after chronic intake of strawberry and honey bioactive compounds and a special care is also devoted to studies regarding the hypocholesterolemic/hypotrigliceridemic potentials of such compounds [17,20,21,26].

**7.**  **Has the focus of your research changed over the years?**

I began investigating the mitochondrial respiratory chain enzymes, their structures and properties, and my first interest was to understand which role antioxidants could have when they interact with some of these components. Gradually, I widened the number and kind of antioxidants and then I begin to focus on the foods where they were contained.

**8.**  **What is the next stage in food research on the role of natural bioactive compounds?**

The next stage, actually, could be that of producing foods with enhanced amounts of some of these compounds. We have patented, for example, a strawberry variety that possess 8-fold the current folate contents of a commercially available strawberry: the idea is to assist foods’ production from farm to fork in order to give the consumers not only a healthy product but even a product that could help and assist consumers in their daily challenge for a healthy diet which could positively have repercussions on the prevention of the most diffused chronic and degenerative diseases.

**9.**  **What are some of the most urgent challenges in treating mitochondrial impairment diseases?**

It could seem crazy to say that the main problem of mitochondrial diseases is that they are terrible diseases which affect only a limited number of patients but it is true in the sense that in many countries they are not perceived as an actual and serious problem for Public Health. Therefore, the research grants are terribly limited and the efforts depend upon researchers of good will who usually dedicate part of their time and grants to also study mitochondrial diseases. It is absolutely required to dedicate more efforts to studying them because they could present shared mechanisms which could be of help in several different situations [7,25,27,28].

**10.**  **Which recently developed technologies may help with this?**

I came back to investigate mitochondria, a few years ago, when SeaHorse made available a technology which allows us to evaluate and to monitor both glycolisis and mitochondrial respiratory activity in whole living cells and in tissue slices. I think that the technologies which will affect our investigations most in the next 5 years will be 3D cell-cultures (with tissue-printers) and NanoString: I personally planned to change most of our investigation using 3D-tissue printers gradually within the next months; unfortunately, I cannot afford a NanoString device and I hope that somebody reading this interview can support our lab in this sense: it would be a strategic investment.

**11.**  **Where do you see the potential of “bioactive compounds in berries” to human health?**

It has been widely demonstrated that berries’ bioactive compounds affect several metabolic routes and, perhaps, the most intriguing results are the last ones which suggest a possibility for them to actively prevent the onset and development of some kind of cancers. We have obtained striking evidence and we are now in the process of fierce discussion with reviewers of some journals.

**12.**  **Do you have some advice for other groups who are thinking of embarking on nutrition and health projects?**

The field is very competitive and only new, innovative and creative ideas may have the chance to survive: be rigorous but imaginative.

**13.**  **Thank you for your answers to the above questions on your research! It is well known that you are the Editor-in-Chief and an Editorial Board Member of several open access journals. Do you remember when the idea of becoming involved in open access publishing first occurred to you?**

I was curious to see how an Open Access journal worked: firstly, it was just a curiosity but I immediately remain really astonished to see the great work behind it. My previous expertise in the editorial field regarded traditional journals; therefore, I found this new challenge exciting. I began about four years ago and, actually, in the meantime the Open Access world has experimented to undertake the most incredible evolution and improvements. Nowadays, in addition, all the main important traditional Publishers are offering full Open Access Journals in addition to Open Access possibilities in the historical journals.

**14.**  **How did this come about?**

I was contacted several times but discharged most of the emails as SPAM. Once, the offer to guest edit a special issue attracted my attention and asked for additional information.

**15.**  **What do you think the idea of open access contributes to the publishing world?**

Open Access deeply contributed to the development of publishing world and will be more and more important in the next few years: an irrefutable movement towards Open Access devices is gaining in all the knowledge areas and Open Access will very suddenly represent the most diffused choice for researchers. In this moment, probably, too many companies with limited experience in the scientific editorial fields have launched new (too many and uncontrolled) journals. I think that, if there will be a way to control SPAM offers, Open Access could actually represent the most important contribution to publishing world.
